# Comparative Analysis of Palatal Depth and Nasal Septum Deviation in Patients with and without Maxillary Canine Impaction: A Cone Beam Computed Tomography Study

**DOI:** 10.31661/gmj.v13iSP1.3694

**Published:** 2024-12-31

**Authors:** Neda Shahraki, Fatemeh Keikhaee, Fatemeh Kahnemuee

**Affiliations:** ^1^ Department of Orthodontics, Oral and Dental Disease Research Center, Zahedan University of Medical Sciences, Zahedan, Iran; ^2^ Department of Orthodontics, Zahedan Dental School, Zahedan University of Medical Science, Zahedan, Iran

**Keywords:** Tooth, Impacted, Canine, Nasal Septum, Palate, Hard

## Abstract

**Background:**

The primary aim of this study was to compare palate depth and nasal septum deviation between patients with unilateral and bilateral buccal and palatal impactions of maxillary canines and those without impaction.

**Materials and Methods:**

This cross-sectional study examined CBCT images of 60 patients from a private radiology archive, divided into four subgroups of 10 patients each with unilateral or bilateral buccal and palatal impactions, and a control group of 20 patients without impaction. Nasal deviation was assessed by measuring the distance of the maximum convexity of the deviated septum from the midsagittal plane in the coronal CBCT cut. Palate depth (PD) was measured as the perpendicular line from the middle of the axis connecting the mesiopalatal cusp of the first molar to the hard palate. Measurements were performed using NNT 16.3.1 software and validated by radiology and orthodontics specialists. Independent t-tests were used for statistical comparisons.

**Results:**

There were no significant differences in palate depth (p 0.05) or nasal septum deviation (P 0.05) between the control group and patients with unilateral or bilateral buccal and palatal impactions of maxillary canines.

**Conclusion:**

The study found no significant differences in palate depth and nasal septum deviation between patients with and without maxillary canine impaction, suggesting that impaction does not significantly affect these anatomical features. Further research is recommended to explore these findings in larger populations.

## Introduction

Canine impaction is defined by the failure of the canine tooth to erupt within the expected time frame, often due to its positioning below the alveolar bone [1][2]. Maxillary canine impaction is the second most common dental impaction, affecting approximately 2% of the population, with a higher prevalence in females and a greater incidence in the maxilla compared to the mandible [3][4]. These impacted canines can deviate from their normal path, presenting as either buccal or palatal impactions, each associated with distinct etiologies and clinical implications. Palatal impactions are twice as common in females as males and are significantly more prevalent in the maxilla [5][6].

Factors contributing to maxillary canine impaction include disturbances in the dental lamina, early canine development in the maxilla, and minor forms of cleft lip and palate, such as submucous cleft palate [5][6]. Palatially impacted canines often exhibit an autosomal dominant inheritance pattern with low penetrance and variable expressivity. While palatal impactions are generally associated with an excess of space in the dental arch, buccal impactions typically result from a lack of sufficient space for eruption [7]. The precise etiology of palatal canine impactions remains debated. Still, the adjacent lateral incisor is thought to play a crucial role, either due to genetic factors affecting both teeth or its positional influence on the canine’s eruption pathway [4][8].

Understanding the anatomical variations associated with these impactions, such as palatal depth and nasal septum deviation, is critical for diagnosis and treatment planning in orthodontics [9][10]. Impacted canines can influence adjacent structures, leading to orthodontic issues like altered arch width and deep palates [11][12]. However, specific studies evaluating the relationship between maxillary canine impaction and alterations in palatal depth and nasal septum deviation are limited. Additionally, while cone-beam computed tomography (CBCT) provides precise visualization of impacted teeth and adjacent structures, previous studies often lack a comparative analysis involving control groups without impaction, making it difficult to establish definitive clinical correlations [13][14].

As previous studies on maxillary canine impaction have been limited by a lack of comparative analysis with control groups and have not specifically investigated the Iranian population, we aimed to compare the palatal depth and nasal septum deviation in patients with and without maxillary canine impaction using CBCT in an Iranian population; this study is novel in that it provides a comprehensive analysis of these anatomical features in a specific ethnic group, which may exhibit distinct characteristics due to genetic and environmental factors, and sheds light on the relationship between maxillary canine impaction and adjacent structures in this population.

## Materials and Methods

This cross-sectional study was conducted using cone-beam computed tomography (CBCT) images from a private radiology archive. Ethical approval was obtained from the Institutional Review Board of Zahedan University of Medical Sciences (IR.ZAUMS.REC.1402.371). The study adhered to the ethical principles of the Declaration of Helsinki, ensuring the confidentiality of all patient information.

CBCT images were retrospectively collected from patients who visited a private radiology clinic between January 2018 and December 2022. Patients were included if they had unilateral or bilateral buccal and palatal impactions of the maxillary canine. Exclusion criteria included patients with edentulous ridges, cleft lip and palate, craniofacial syndromes, previous orthodontic treatment, or orthognathic surgery.

The sample size was determined using the formula:


n = \frac{(Z_{\alpha/2} + Z_{\beta})^2 \times 2 \times \sigma^2}{d^2}

Assuming a confidence level of 95% (α = 0.05) and a critical value of 1.96, the minimum sample size was calculated as 7 per group, and 10 patients were included per subgroup for a total of 60 patients: 20 patients with unilateral impaction (10 buccal, 10 palatals), 20 with bilateral impaction (10 buccal, 10 palatals), and 20 controls without impaction. Patients were randomly selected and matched to ensure similar distributions of gender and age across the groups. If initial random selection resulted in significant differences in gender or age, adjustments were made to balance these characteristics.

CBCT scans were acquired using the NEW TOM Model (GIANO HR, voxel size: 300-68 microns) with a field of view (FOV) ranging from 10x8 cm to 16x18 cm depending on scan mode (Prime, Advanced, or Professional 3D). Exposure times varied from 26 to 306 seconds. The scans were analyzed using NNT-16.3.1 software. Measurements were performed on coronal CBCT cuts to assess nasal septum deviation and palate depth. Nasal septum deviation was defined as the distance from the maximum convexity of the deviated septum to the midsagittal plane. Palate depth was measured as the perpendicular distance from the axis connecting the mesiopalatal cusp of the first molar to the hard palate. All measurements were reviewed and approved by a radiologist and an orthodontic specialist.

Measurements were conducted by two trained orthodontists and verified by a radiologist specializing in CBCT analysis. Calibration between operators was performed to minimize performance bias and ensure measurement consistency across all steps of the protocol.

After collecting the data and calculating the mean and standard deviation in each subgroup based on the presented table, SPSS-22 software (SPSS Inc, Chicago, 1L, USA) was used for statistical analysis. Also, the means palate depth and the nasal septum deviation in the buccal and palatal impaction group and the control group, (both unilateral and bilateral impaction types) were compared using a t-test.

The significance level for all data was considered at 0.05.

## Results

**Table:** Table1. Palate Depth (PD) and Nasal Septum Deviation (NSD) measurement results in 5 investigated sub-spheres

**Variables**	**Central and dispersion ** **indices of groups **	**Mean ±SD (mm) **	**Median **	**IQR**	**Min**	**Max**	**P-value for the test **
	unilateral buccal canine impaction	18.98±2.20	19.15	2.90	15.60	23.21	
	bilateral buccal canine impaction	20.39±3.25	20.80	1.80	12.60	25.50	
**PD**	unilateral palatal canine impaction	20.29±1.96	20.10	2.70	17.60	24.50	0.35
	bilateral palatal canine impaction	19.74±3.55	19.45	6.35	15.30	26.40	
	Patients without any maxillary canine impaction(control)	20.80±2.93	21.25	4.32	14.10	25.70	
	unilateral buccal canine impaction	0.61±1.58	0.00	0.28	0	5.00	
	bilateral buccal canine impaction	1.85±2.19	1.25	3.35	0	6.50	
**NSD**	unilateral palatal canine impaction	3.09±2.76	4.25	5.33	0	6.90	0.09
	bilateral palatal canine impaction	2.60±2.08	2.80	4.63	0	5.70	
	Patients without any maxillary canine impaction(control)	1.22±1.62	0.40	2.53	0	5.90	

**IQR:** Interquartile Range

In the present study, 60 CBCT samples were investigated. Among them, 60 samples, 24 females and 16 males were in the study group and 8 males and 12 females were in the control group. The age range of people whose CBCT sample was used was from 9 to 55 years old (with a mean age of 18.25 years) in the study group and from 8 to 49 years old (with a mean age of 30 years) in the control group.

First, the Shapiro-Wilk test was performed. Its results showed that the data distribution in all groups does not follow the normal distribution (P<0.05). Therefore, the Kruskal-Wallis test was used to compare the groups. Table-1 presents the measurement results for palate depth (PD) and nasal septum deviation (NSD) in five subgroups: unilateral buccal canine impaction, bilateral buccal canine impaction, unilateral palatal canine impaction, bilateral palatal canine impaction, and a control group without any maxillary canine impaction. For PD, the mean values range from 18.98 mm (unilateral buccal) to 20.80 mm (control), with medians ranging from 19.15 mm to 21.25 mm. The interquartile ranges (IQR) vary from 1.80 mm to 6.35 mm, indicating the spread of the middle 50% of the data. For NSD, the mean values range from 0.61 mm (unilateral buccal) to 3.09 mm (unilateral palatal), with medians ranging from 0.00 mm to 4.25 mm. The IQRs for NSD range from 0.28 mm to 5.33 mm. The Kruskal-Wallis test showed no statistically significant differences in PD (p = 0.35) or NSD (p = 0.09) among the subgroups.

For the unilateral buccal canine impaction subgroup, the median PD was 19.15 mm with a mean of 18.98 ± 2.20 mm, and the median NSD was 0 mm with a mean of 0.61 ± 1.58 mm. The control group had a median PD of 21.25 mm with a mean of 20.80 ± 2.93 mm, and a median NSD of 0.40 mm with a mean of 1.22 ± 1.62 mm. The P-values for the PD and NSD comparisons between the unilateral buccal subgroup and the control group were 0.09 and 0.21, respectively, indicating no statistically significant differences. For the bilateral buccal canine impaction subgroup, the median PD was 20.80 mm with a mean of 20.39 ± 3.25 mm, and the median NSD was 1.25 mm with a mean of 1.85 ± 2.19 mm. The control group had a median PD of 21.25 mm with a mean of 20.80 ± 2.93 mm, and a median NSD of 0.40 mm with a mean of 1.22 ± 1.62 mm. The P-values for the PD and NSD comparisons between the bilateral buccal subgroup and the control group were 0.71 and 0.53, respectively, indicating no statistically significant differences. For the unilateral palatal canine impaction subgroup, the median PD was 20.10 mm with a mean of 20.29 ± 1.96 mm, and the median NSD was 4.25 mm with a mean of 3.09 ± 2.76 mm. The control group had a median PD of 21.25 mm with a mean of 20.80 ± 2.93 mm, and a median NSD of 0.40 mm with a mean of 1.22 ± 1.62 mm. The P-values for the PD and NSD comparisons between the unilateral palatal subgroup and the control group were 0.45 and 0.13, respectively, indicating no statistically significant differences. For the bilateral palatal canine impaction subgroup, the median PD was 19.45 mm with a mean of 19.74 ± 3.55 mm, and the median NSD was 2.80 mm with a mean of 2.60 ± 2.08 mm. The control group had a median PD of 21.25 mm with a mean of 20.80 ± 2.93 mm, and a median NSD of 0.40 mm with a mean of 1.22 ± 1.62 mm. The P-values for the PD and NSD comparisons between the bilateral palatal subgroup and the control group were 0.35 and 0.10, respectively, indicating no statistically significant differences.

## Discussion

This study aimed to investigate palate depth and nasal septum deviation in patients with maxillary canine impaction, comparing various subgroups (unilateral buccal, unilateral palatal, bilateral buccal, and bilateral palatal) to a control group without impaction. Our findings revealed no significant differences in palate depth or nasal septum deviation between the impaction groups and the control group, suggesting that these anatomical features may not be influenced by the presence of canine impaction.

Previous research by Schindel and Duffy (2007) has demonstrated that impacted canines, particularly those in the palatal position, are often associated with deeper palatal vaults compared to non-impacted counterparts. Their study suggested that palatal morphology might play a role in the development of impaction, potentially serving as both a contributing factor and a consequence of impaction [15]. This aligns with our findings, which showed variable palatal depths among different impaction types, although not statistically significant.

The lack of difference in palate depth aligns with findings by Elmarhoumy (2023) and Sharhan et al. (2022), who also reported no significant variations between patients with canine impaction and control groups [16][17]. In contrast, some studies have found notable differences. For instance, Yassaei et al. (2022) observed that maxillary arch width and palate volume were significantly reduced on the impaction side compared to the non-impaction side, highlighting potential variations in specific anatomical measurements that were not evident in our broader comparison [13]. Similarly, Genc et al. (2023) and Sobhani (2023) identified smaller palate dimensions in impaction groups compared to controls, particularly in cases of palatal impaction, suggesting that variations in study design, measurement techniques, and sample sizes may influence these conflicting results [18][19].

Studies have shown varying results regarding the association between maxillary canine impaction and nasal septum deviation. For instance, Erhamza et al. (2021) found that maxillary canine impaction might be linked to altered craniofacial structures, including nasal septum deviation, suggesting that the impaction may affect overall facial symmetry [20]. However, conflicting evidence was presented by Tassoker et al. (2020), who reported no significant correlation between nasal septum deviation and maxillary canine impaction, highlighting the need for further research to clarify this relationship [21].

Regarding nasal septum deviation, previous studies by Kucukkaraca et al. (2023) and Elmarhoumy (2023) indicated higher levels of septum deviation in patients with canine impaction compared to controls [16][22]. Elmarhoumy (2023) reported septum deviation in 60% of patients with palatal impaction and 80% in those with labial impaction, compared to only 10% in controls, suggesting a potential association between impaction and nasal deviation [16]. However, our results did not replicate these findings, highlighting the need for further research to clarify the relationship between canine impaction and nasal septum positioning.

The impact of maxillary canine impaction on palatal depth in Iranian patients has been explored in several studies, revealing mixed findings. Yassaei et al. (2022) found that while maxillary canine impaction is significantly associated with a reduction in maxillary arch width (P < 0.001), there was no significant correlation between canine impaction and palatal depth (R = 0.15, P-value = 0.326). However, the study did highlight a significant correlation between canine impaction and palatal volume (R = 0.728, P-value < 0.001), suggesting that the volume of the palate is more affected than its depth [23]. In contrast, Farhadifard et al. (2024) observed that canine impaction did not result in statistically significant differences in palatal depth when compared to the control group, despite significant changes in other maxillary dimensions such as arch circumference, arch length, and intermolar width [24]. Similarly, Fattahi et al. (2023) reported no statistically significant differences in palatal depth or palatal height index between patients with palatal canine impaction and a matched control group [25]. These findings suggest that while maxillary canine impaction can influence various aspects of maxillary arch morphology, its effect on palatal depth is less pronounced and may not be a consistent feature across different patient populations. These confirmed our study findings.

The limited number of studies examining nasal septum deviation in relation to maxillary canine impaction restricts the ability to draw definitive conclusions. The septum’s formation occurs earlier in life compared to the timing of canine impaction, implying that impaction may not directly affect septal positioning. Differences in the anatomical and developmental timing of these structures warrant cautious interpretation of any associations observed.

A key strength of our study is the comprehensive analysis of various impaction types (unilateral vs. bilateral and buccal vs. palatal), allowing for a more detailed understanding of anatomical variations. Additionally, our sample size was adequate relative to other studies in the field, enhancing the reliability of our findings. However, the variability in measurement methods and sample demographics across studies highlights the need for standardized protocols in future research.

## Conclusion

Based on the investigations and statistical analysis in this study, the following results were obtained: There is no difference between the control group without impaction and the patients with unilateral and bilateral buccal and palatal impactions in terms of palatal depth. Furthermore, there is no difference between the control group without impaction and the patients with unilateral and bilateral buccal and palatal impaction in terms of nasal septum deviation. It is recommended that more studies be conducted to measure other dimensions of the mouth and nose cavity that can be affected or cause canine impaction to clarify its etiology as much as possible.

## Conflict of Interest

Authors declare no conflict of interest.
